# Postmortem Lividity Color Change During Rewarming Following Cold Storage: A Case Report of Drowning

**DOI:** 10.7759/cureus.88687

**Published:** 2025-07-24

**Authors:** Ikuto Takeuchi, Haruka Ikeda, Motoo Yoshimiya, Atsushi Ueda, Yu Kakimoto

**Affiliations:** 1 Department of Forensic Medicine, Tokai University School of Medicine, Isehara, JPN

**Keywords:** cherry-red livor mortis, forensic autopsy, postmortem lividity, refrigeration, rewarming

## Abstract

Postmortem lividity typically presents as purplish discoloration due to the accumulation of deoxygenated blood in dependent body regions after death. However, certain pathological and environmental conditions can alter its appearance, occasionally resulting in a cherry-red hue that may mislead forensic interpretation. We report a case of drowning in which postmortem lividity appeared cherry-red following cold storage but gradually turned purplish during surface rewarming before autopsy. This observation illustrates the reversible, temperature-dependent behavior of lividity dominated by oxygenated hemoglobin (O₂Hb), contrasting with the stable coloration seen in carbon monoxide (CO) poisoning. Understanding such changes is critical in forensic evaluations to avoid diagnostic pitfalls associated with livor mortis coloration.

## Introduction

Postmortem lividity, also known as livor mortis, typically develops within 30 minutes to two hours after death and becomes fixed within eight to 12 hours [1]. The typical purplish hue arises from the accumulation of deoxygenated hemoglobin in dependent blood vessels [2]. However, the color of lividity can vary significantly depending on physiological and environmental factors. In certain contexts, it may appear bright red or cherry-red.

This atypical appearance has been reported in cases of carbon monoxide poisoning, cyanide poisoning, hypothermia, and prolonged postmortem refrigeration, and may serve as an important forensic clue [3-7]. Nevertheless, the physiological mechanisms behind these color changes, particularly their reversibility, are not fully understood.

Here, we present a rare case of drowning in which cherry-red lividity was observed following refrigerated storage. Notably, this color gradually shifted to purplish during surface rewarming prior to autopsy. This dynamic transformation provides novel insight into the temperature-sensitive properties of oxygenated hemoglobin (O₂Hb) in postmortem tissue and emphasizes the importance of considering environmental history during forensic examination.

Drowning is an asphyxial condition primarily caused by aspiration of water into the airways. In forensic contexts, drowning cases often involve full-body immersion, during which water temperature and movement can affect the position of the body and complicate the assessment of postmortem lividity [8]. These factors should be considered when interpreting hypostasis patterns in water-related deaths.

## Case presentation

The decedent was a woman in her 20s found floating in the sea under unknown circumstances and pronounced dead at the scene. The estimated postmortem interval at the time of discovery was approximately one day. According to meteorological data, the ambient air temperature was 21℃, and the seawater temperature was 18℃. The case was referred to the forensic department of our university by the relevant maritime authorities for a judicial autopsy.

Following recovery, the body was refrigerated at approximately 4℃ for five days. At the time of autopsy, the rectal temperature measured 5.0℃, while the room temperature was 18.7℃. The skin and subcutaneous tissues were notably firm and rigid, suggesting substantial surface cooling. This was presumed to have resulted from the overcooling of the body surface during transportation from the storage facility to our department on the day of autopsy.

**Figure 1:**
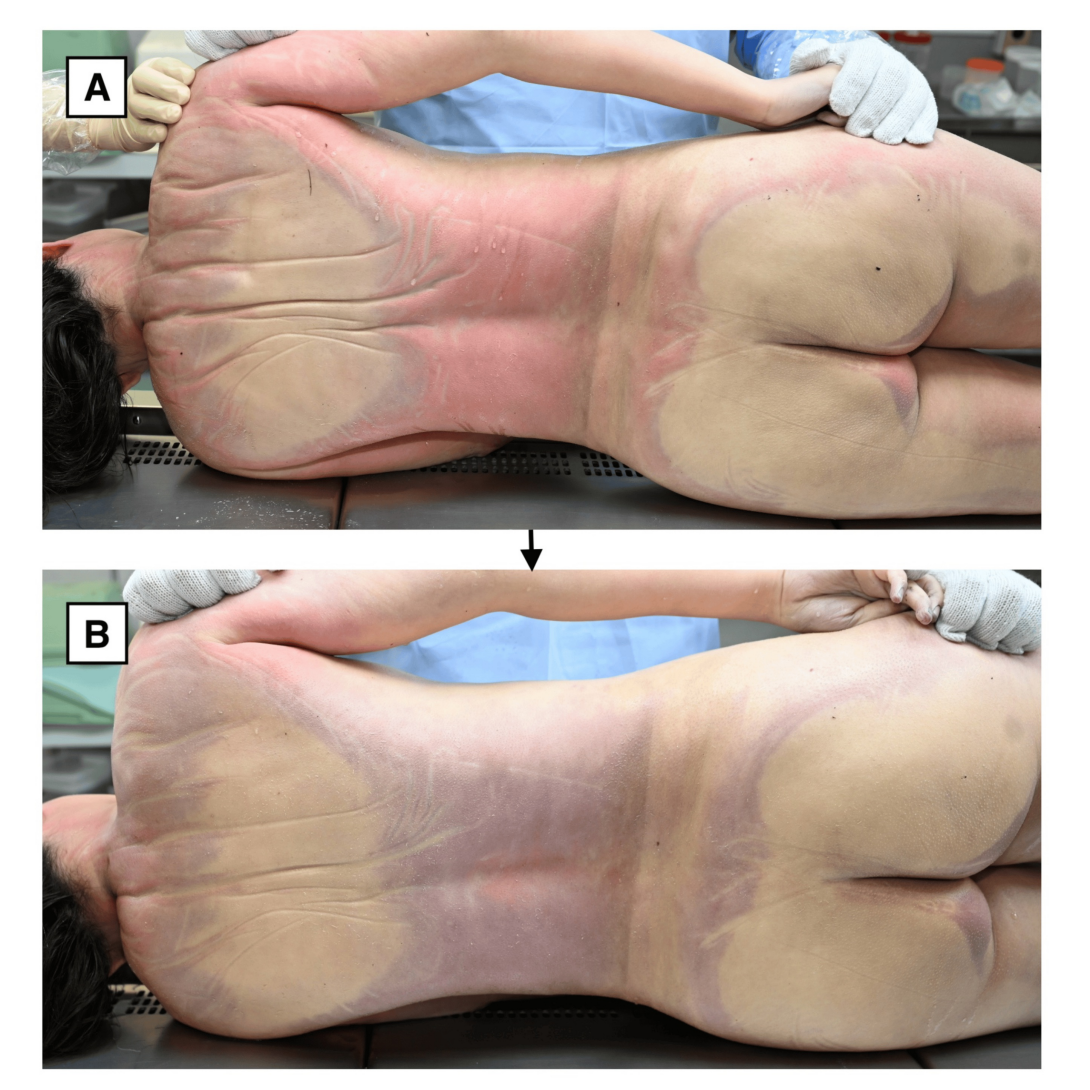
Postmortem lividity color change during rewarming (A) Cherry-red discoloration of the posterior lividity before surface rewarming. (B) Purplish discoloration observed 22 minutes after rewarming. The images illustrate the reversible and temperature-dependent transformation of postmortem lividity dominated by oxygenated hemoglobin (O₂Hb).

Due to difficulty in standard dissection caused by extreme firmness of the superficial tissues, surface rewarming was performed using warm water to facilitate tissue softening. During this process, a striking change in the color of the posterior lividity was observed. Initially, lividity appeared cherry-red (Figure 1A). As rewarming progressed, the color gradually shifted to a purplish hue (Figure 1B).

The interval between the two photographs documenting this transformation was 22 minutes, clearly demonstrating the dynamic and temperature-dependent nature of the phenomenon. After rewarming, the softening of the body surface became evident, further supporting the hypothesis of significant pre-autopsy surface overcooling.

Subsequently, a full autopsy was performed. Frothy fluid was present in the trachea, and the lungs were markedly overdistended, voluminous, and edematous. No significant findings were observed other than those consistent with drowning. Histological examination of the lungs revealed pulmonary edema with frothy fluid, supporting the diagnosis. Toxicological testing, including alcohol and common drugs of abuse, showed negative results. Based on the totality of findings, the cause of death was determined to be asphyxia due to drowning.

## Discussion

Postmortem lividity usually appears within 30 minutes to two hours after death and becomes fixed within eight to 12 hours [1]. Its characteristic purplish hue reflects the predominance of deoxygenated hemoglobin in static postmortem blood [2]. However, certain pathological or environmental conditions may produce lividity with bright red or cherry-red coloration, which can serve as important indicators in forensic diagnostics.

Cherry-red lividity has been documented in cases of carbon monoxide poisoning, cyanide poisoning, hypothermia, and postmortem refrigeration [3-7]. In carbon monoxide poisoning, carboxyhemoglobin (COHb) is formed as carbon monoxide binds strongly to hemoglobin, producing the well-known cherry-red appearance [3-5]. This finding is often regarded as a hallmark sign of CO poisoning. However, not all CO poisoning cases present with this discoloration, possibly due to peripheral vasoconstriction that limits COHb-rich blood distribution to the skin [9]. Notably, once formed, COHb is highly stable and does not dissociate or degrade under various storage temperatures, including +20℃, +4℃, and even -70℃, for extended periods of up to 12 weeks [10].

In contrast, cyanide poisoning leads to impaired cellular oxygen utilization, elevating venous oxygen saturation and resulting in cherry-red lividity dominated by oxygenated hemoglobin (O₂Hb) [6]. Although the mechanisms differ, the visual presentation can resemble that of CO poisoning. To date, no reports have described temperature-induced color shifts in cyanide-related lividity.

Postmortem refrigeration is another recognized cause of cherry-red lividity, attributed to passive oxygen diffusion into pooled blood, increasing hemoglobin oxygen saturation [7]. This phenomenon is temperature-dependent: lower temperatures increase both the solubility of oxygen and the oxygen-binding affinity of hemoglobin. Upon rewarming, these properties reverse-oxygen solubility decreases, and hemoglobin may release bound oxygen, potentially causing a transition from cherry-red to purplish discoloration [11].

Furthermore, the capacity for postmortem reoxygenation may diminish over time, limiting this phenomenon to early postmortem periods [12]. The present case offers rare photographic documentation of reversible lividity color change during rewarming, a finding not widely reported in forensic literature.

Unlike COHb-associated lividity, which remains stable regardless of environmental conditions [10], lividity dominated by O₂Hb appears to be temperature-sensitive [11]. This case underscores the importance of considering environmental context, particularly refrigeration and rewarming, in the interpretation of livor mortis. Recognizing this variability is essential to avoid misattributing cherry-red discoloration to exogenous poisoning, especially in cases involving cold storage.

Exposure to cold environments is a recognized factor contributing to the development of cherry-red lividity. Spectrophotometric studies have demonstrated that when the skin temperature drops to approximately 10℃, a qualitative change occurs in the skin reflectance spectrum, indicating a shift in livor mortis color from dark red to cherry red. This phenomenon is consistent with temperature-dependent oxygenation of hemoglobin under low-temperature conditions [7].

In the present case, the seawater temperature at the time of discovery was 18℃, which is lower than normal body temperature and potentially capable of inducing hypothermia with prolonged exposure. However, this level of cooling alone is unlikely to have produced the observed lividity. In contrast, postmortem refrigeration at approximately 4℃ for five days was likely the primary factor contributing to the cherry-red discoloration.

Additionally, it has been reported that livor mortis tends to be indistinct in drowning victims due to unstable body positioning caused by floating or water currents [8]. Lividity is generally considered highly mobile within the first 12 hours after death, but may remain displaceable for up to approximately 48 hours [13]. In this case, the body was recovered approximately one day after death, suggesting that lividity had not yet become fixed. Therefore, stabilization of body position during refrigeration, combined with cold-induced hemoglobin oxygenation, likely resulted in the consistent cherry-red lividity observed on the dorsal surface.

In this case, clear contact pallor was noted on the upper back and buttocks, which likely reflects the maintenance of a supine position during refrigerated storage. Furthermore, prior to rewarming, a purplish discoloration was observed partially surrounding these areas of contact pallor.

Before dorsal surface observation, the body had been placed in a supine position on a metal autopsy table at a room temperature of 18.7℃. It is possible that localized surface warming had begun at the points of contact, leading to regional increases in temperature around the margins of the pallor zones. This may have facilitated localized oxygen dissociation and contributed to the appearance of purplish discoloration even before active rewarming began.

## Conclusions

We report a rare case in which postmortem lividity initially appeared cherry-red following refrigerated storage and shifted to a purplish hue during rewarming. This reversible change was likely driven by temperature-dependent alterations in oxygenated hemoglobin (O₂Hb) binding dynamics.

Recognition of this phenomenon is crucial in forensic practice, as cherry-red lividity is often associated with carbon monoxide or cyanide poisoning. Understanding the temperature-sensitive behavior of livor mortis coloration can help forensic pathologists avoid misinterpretation and ensure accurate cause-of-death assessments, particularly in refrigerated bodies.

## References

[1] Henssge C, Madea B (2004). Estimation of the time since death in the early post-mortem period. Forensic Sci Int.

[2] Bohnert M, Weinmann W, Pollak S (1999). Spectrophotometric evaluation of postmortem lividity. Forensic Sci Int.

[3] Schäffer B, Peschel O (2018). The differential diagnosis of light-red livor mortis. Dtsch Arztebl Int.

[4] Nielsen PR, Gheorghe A, Lynnerup N (2014). Forensic aspects of carbon monoxide poisoning by charcoal burning in Denmark, 2008-2012: an autopsy based study. Forensic Sci Med Pathol.

[5] Ruas F, Mendonça MC, Real FC, Vieira DN, Teixeira HM (2014). Carbon monoxide poisoning as a cause of death and differential diagnosis in the forensic practice: a retrospective study, 2000-2010. J Forensic Leg Med.

[6] Parker-Cote JL, Rizer J, Vakkalanka JP, Rege SV, Holstege CP (2018). Challenges in the diagnosis of acute cyanide poisoning. Clin Toxicol (Phila).

[7] Bohnert M, Schulz K, Belenkaia L, Liehr AW (2008). Re-oxygenation of haemoglobin in livores after post-mortem exposure to a cold environment. Int J Legal Med.

[8] Lunetta P, Zaferes A, Modell J (2014). Establishing the cause and manner of death for bodies found in water. Drowning.

[9] Carson HJ, Esslinger K (2001). Carbon monoxide poisoning without cherry-red livor. Am J Forensic Med Pathol.

[10] Wachsmuth NB, Bajaa B, Wachsmuth C, Schmidt WF (2025). Stability of carboxy-hemoglobin during storage at different temperatures. Drug Test Anal.

[11] Weber RE, Campbell KL (2011). Temperature dependence of haemoglobin-oxygen affinity in heterothermic vertebrates: mechanisms and biological significance. Acta Physiol (Oxf).

[12] Watchman H, Walker GS, Randeberg LL, Langlois NE (2011). Re-oxygenation of post-mortem lividity by passive diffusion through the skin at low temperature. Forensic Sci Med Pathol.

[13] Vanezis P, Trujillo O (1996). Evaluation of hypostasis using a colorimeter measuring system and its application to assessment of the post-mortem interval (time of death). Forensic Sci Int.

